# Layer-by-Layer Coating of MK-40 Heterogeneous Membrane with Polyelectrolytes Creates Samples with Low Electrical Resistance and Weak Generation of H^+^ and OH^−^ Ions

**DOI:** 10.3390/membranes11020145

**Published:** 2021-02-20

**Authors:** Kseniia Tsygurina, Olesya Rybalkina, Konstantin Sabbatovskiy, Evgeniy Kirichenko, Vladimir Sobolev, Ksenia Kirichenko

**Affiliations:** 1 Physical Chemistry Department, Kuban State University, 149 Stavropolskaya St., 350040 Krasnodar, Russia; kseniya_alx@mail.ru (K.T.); olesia93rus@mail.ru (O.R.); 2 Frumkin Institute of Physical Chemistry and Electrochemistry RAS, 31 Leninsky Prospect, 119071 Moscow, Russia; sabbat07@mail.ru (K.S.); vsobolev@phyche.ac.ru (V.S.); 3 Department of Public and International Law, Kuban State Agrarian University Named after I.T. Trubilin, 13 Kalinina St., 350004 Krasnodar, Russia; lbcs@yandex.ru

**Keywords:** ion exchange membrane, membrane modification, layer by layer coating, voltammetry, limiting current density, monovalent selectivity

## Abstract

Ion exchange membranes covered with layers of polyelectrolytes of alternating charges are characterized by very high monovalent selectivity. This allows the use of such membranes for electrodialytic fractionation of multicomponent solutions. However, the very existence of the boundary at which differently charged layers come in contact can hinder a membrane’s effectiveness by limiting its ion permeability, raising levels of H^+^ and OH^−^ ions (thus shifting the pH) and increasing the electrical resistance of the membrane, which leads to increased energy consumption. To test how these properties would be changed, we created cheap layer-by-layer-modified membranes based on the heterogeneous MK-40 membrane, on which we adsorbed layers of polyallylamine and sulfonated polystyrene. We created samples with 3, 4, and 5 layers of polyelectrolytes and characterized them. We showed that the application of layers did not decrease the efficiency of the membrane, since the electrical resistance of the modified samples, which increased after application of the first oppositely charged layer, declined with the application of the following layers and became comparable to that of the substrate, while their limiting current density was higher and the shift of pH of treated solution was low in magnitude and comparable with that of the substrate membrane.

## 1. Introduction

Electrodialysis with ion exchange membranes is one of the main methods of water treatment [1]. Using electrodialysis or hybrid (e.g., when the electrodialysis concentrates the reverse osmosis retentate) installations, it is possible to obtain drinking water or more deeply purified water in regions where water of adequate quality is not available, starting from polluted, brackish, or salt water as the source [2,3,4].

However, there are also tasks for which the main goal is not total demineralization, but rather partial desalination with preservation of some type of ions. For drinking water for humans and farm animals or for irrigation, it is necessary that the water contains a number of ions required for the normal growth and development of living organisms, while at the same time not being excessively salty [5,6]. When stabilizing mining waters, the goal is to remove or separate Ca^2+^ and SO_4_^2−^ ions, which provide sediment if they are simultaneously present in the solution [7,8]. Water containing excessive nitrates cannot be used for drinking, however it can become suitable after nitrate removal [9]. Similar tasks include the extraction of lithium from lake brines, where it must be separated from magnesium [10,11].

Some of these tasks can be reduced to the challenge of separation of singly and polycharged ions. Considering the methods for solving this challenge, it seems promising to create layer-by-layer coated materials with alternating charges of fixed groups, since it has been shown in published studies that the advantages of this method include very high achieved selectivity (in article [12], for example, K^+^/Mg^2+^ selectivity exceeded 1000), the availability of materials due to relatively cheap polyamines [13,14,15,16,17] and polystyrene sulfonate [18,19,20,21] being widely and successfully used, and the simplicity of technique, since it has been demonstrated that even the adsorption from a solution without covalent linking results in remarkable samples [12,15,18].

Layer-by-layer-modified membranes are proposed for electrodialysis fractionation of solutions. To achieve a more energy-efficient process, such membranes should have low resistance, a high transport number of counterions (selectivity), and a high limiting current density of salt ions. Published studies indeed report the excellent performance of the created samples [12,22]. At the same time, earlier works describing deposition of relatively thin layers of polyelectrolyte not done in a layer-by-layer manner, but rather through the creation of one extended layer, reported a modest increase in monovalent selectivity [23] and a change in the functionality of membranes [24]. Let us consider the forming structure to explain the basis behind these findings.

When functional groups with different charge signs contact in a very narrow area, a local electric field with high strength is formed. In the case of traditional bipolar membranes, charged membranes of sufficient thickness are located on both sides of this boundary, and when the external electric field is oriented in such a way that counterions move away from the bipolar boundary, the membrane practically does not transfer salt ions and only generates H^+^ and OH^−^ ions [25,26,27]. In order for the created bilayer membrane to become bipolar, an applied layer thickness as low as 10 μm is sufficient [24].

Bipolar boundaries surrounded by ion exchange layers can be found not only in traditional bipolar membranes, but also in monopolar membranes coated with a polyelectrolyte(s), the functional groups of which are oppositely charged to the functional groups in the membrane bulk [28] and in the layer-by layer materials. However, only for layer-by-layer-modified membranes does high monovalent selectivity occur, with no increase in electrical resistance or the generation of H^+^ and OH^−^ ions. This raises the question as to why the application of a large number of thin layers with alternating charges of functional groups is more preferable for the goals of electrodialysis fractionation than the application of one continuous layer. It also raises the question of whether layer-by-layer-modified ion exchange membranes would experience in some form the changes that occur when a monopolar membrane is coated with a polyelectrolyte to transform it into an asymmetric bipolar membrane, namely if its electrical conductivity and its limiting current density would decrease and the pH shift of the treated solution would increase.

To answer these questions, we carried out a continuing study of the changes of properties of layer-by-layer-modified membranes after the application of each layer. Previously [29], we adsorbed the branched polyethyleneimine (PEI) on the surface of the heterogeneous MK-40 cation exchange membrane. We showed that the created sample retained the limiting current density of the salt counterions but demonstrated increased generation of H^+^ and OH^−^ ions, even in underlimiting current modes. This sample also showed no significant change in monovalent selectivity compared to the original membrane.

In this article, we publish the results for the next stage of the work. The preliminary hypothesis that we tested in this stage was that as the number of adsorbed layers increases, modified membranes remain usable for electrodialysis desalination and fractionation purposes, which in terms of measurable performance means that their electrical conductivity is comparable with the substrate membrane, their limiting current density is equal to or higher than the substrate membrane, and the pH shift of the treated solution is equal to or lower than that of the substrate membrane.

To test the changes in electrochemical properties, we applied a larger number of modifying layers of polyelectrolytes with alternating charges of fixed groups on the same heterogeneous membrane, then recorded the current–voltage curves and the pH difference between the outlet and inlet compared to the desalination channel.

## 2. Materials and Methods

### 2.1. Sample Preparation

We used the MK-40 heterogeneous cation exchange membrane manufactured by Shchekinoazot (Pervomayskiy (Tula Oblast), Russia) as a substrate membrane, as was also done in our previous study [29]. The advantages of this membrane are its low cost, high chemical and mechanical stability, as well as its high selectivity in relation to counterions in comparison with co-ions [30]. The manufacturer states [30] that the membrane is made by hot rolling of powders of KU-2 styrene–divinylbenzene sulfonic cation exchange resin and polyethylene between reinforcing polyamide cloths. As a result, a significant portion of the membrane consists of a durable but non-conductive material, the surface fraction of which is reported to be around 80% [31,32], due to which the membrane’s conductivity and limiting current density value are lower than that of other commercial membranes [33].

There is a technique that can increase the electrical conductivity and limiting current of the MK-40 membrane by coating it with a dispersion of perfluorosulfonic cation exchangers [33]. The authors of the approach suggest that in this way it is possible to improve the properties of this heterogeneous membrane in the electrodialysis purification process to the level of much more expensive homogeneous Nafion membranes and confirm this via comparison of the current–voltage curves. We applied this approach to homogenize the surface in order to increase the active surface on which sorption will be carried out by the mechanism of electrostatic interactions of fixed groups, and also in order to consider this ion exchange membrane homogeneous in the first approximation, as in further mathematical modeling. In the original technique, the heterogeneous membrane is coated with Nafion produced by Dupont (Wilmington, DE, USA). For this purpose, we used its cheaper analogue [34] LF-4SK produced by Plastpolymer (Saint Petersburg, Russia).

We chose sodium polystyrene sulfonate (PSS) as a cation exchange modifier and polyallylamine (PAA) as an anion exchange modifier; both were purchased in solid form from Sigma-Aldrich (Saint Louis, MO, USA). PSS was used in a very large number of previous studies on the monovalent selectivity of layer-by-layer polyelectrolyte assemblies [18,19,20,21], and according to our observations, it is the most common modifier for its role; PAA or PEI was used in the same works as an anion exchange polyelectrolyte [13,14,15,16,17]. We agree with the opinion expressed in several sources, for example in [12], which suggested the electrostatic interaction of fixed groups as a mechanism for attachment of new layers to the surface (electrostatic self-assembly). Our previous step was implemented with PEI and the resulting membrane had some undesired properties, so we tested if replacing it with weaker basic polyamine, and hence lowering the density of positive charges, will yield a better result.

**Figure 1:**
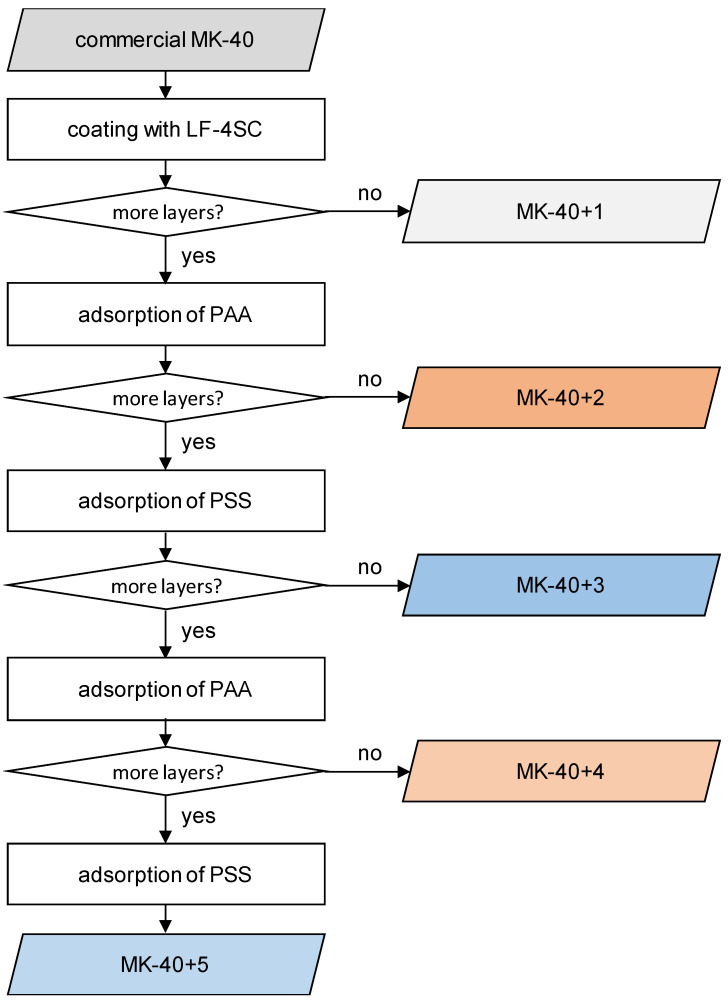
The algorithm used to create modified samples. MK-40 is a brand of commercial heterogeneous cation exchange membranes. The numbers in denotations show the amount of applied layers. The membranes for which the upper layer was formed by PAA (polyallylamine) and was assumed to be positively charged are marked in red. The membranes for which the upper layer was formed by PSS (polystyrene sulfonate) and was assumed to be negatively charged are marked in blue.

An algorithm of actions for the creation of samples with an increasing number of polyelectrolyte layers with alternating charge signs of fixed groups is shown in Figure 1. The procedure consisted of the following stages:

(1) Commercial MK-40 membrane was equilibrated with the saline solution in which the measurements were to be carried out. To do this, the membrane sheet purchased from Shchekinoazot in dry form was cut into samples according to the size of the experimental installations, which were then placed in a concentrated NaCl solution (purchased in solid form at analytical grade from Vekton Ltd. (Saint Petersburg, Russia)) and kept for 1 h. After this, the solution was replaced with 1 M NaCl and kept for 1 h, then the solution was replaced with 0.5 M NaCl and kept for 1 h, then the membranes were placed in 0.02 M NaCl, after which they were kept, periodically replacing the solution, until the electrical conductivity of the equilibrating solution became constant;

(2) On one side of the membrane, the working window of the membrane was covered with an LF-4SK homogenizing layer. For this, the membrane was removed from the solution, wiped with filter paper, fixed with glue tape to the bottom of the Petri dish in such a way that a working window of at least 2 × 2 cm^2^ remained free, and a 7% dispersion of LF-4SK in low molecular weight aliphatic alcohols was spread over the surface with a laboratory spatula to create an approximately even coating layer. The sample was kept in air for half an hour to evaporate the alcohols and solidify the layer. Following the chosen denotation method, this membrane should be denoted as MK-40+1, however the membrane denoted below refers not to the MK-40+1 prepared in this batch (the properties of which were not studied separately), but to the membrane previously modified in this way. This membrane had a small difference in the modification procedure—the modified window was 2.2 × 2.2 cm^2^;

(3) In a Petri dish with an attached membrane, 100 mL of a PAA solution with a concentration of 1 g/L was poured and kept for 1 h. After this, the membrane was washed with working NaCl solutions to remove the unabsorbed polymer. At this stage, some of the membranes were detached and placed in working solutions. Following the designations adopted in a previous article [29], these samples were marked as MK-40+2. At a previous stage of the experiment, we also created a MK-40+2 membrane, however the top layer was not a polyallylamine but rather a polyethyleneimine (purchased in solid form from Sigma-Aldrich Saint Louis, MO, USA), with the rest of the procedure being the same;

(4) Samples subject to further modification were subjected to 100 mL of a 1 g/L PSS solution and kept for 1 h, then washed with working NaCl solutions. The samples whose modification stopped at this stage were returned to the working solutions and further designated as MK-40+3;

(5) The adsorption of PAA was repeated as in step 3. The membranes for which the modification ended at this point were further designated as MK-40+4;

(6) Finally, PSS adsorption was repeated in the same manner as in step 4 to create MK-40+5 samples. One sample of this membrane was equilibrated with a 0.015 M NaCl + 0.0075 M CaCl_2_ (purchased in solid form from Vekton Ltd., Saint Petersburg, Russia) solution and was used for electrodialysis of the mixed solution.

### 2.2. Thickness Measurement

Thickness values of swollen samples equilibrated with 0.02 M NaCl solution were measured using a MKC-25 0.001 micrometer (Micron Ltd., Moscow, Russia). For each sample, the thickness was calculated as the average of 10 measurements at points relatively uniformly distributed over the modified window.

### 2.3. Current–Voltage Curves

**Figure 2:**
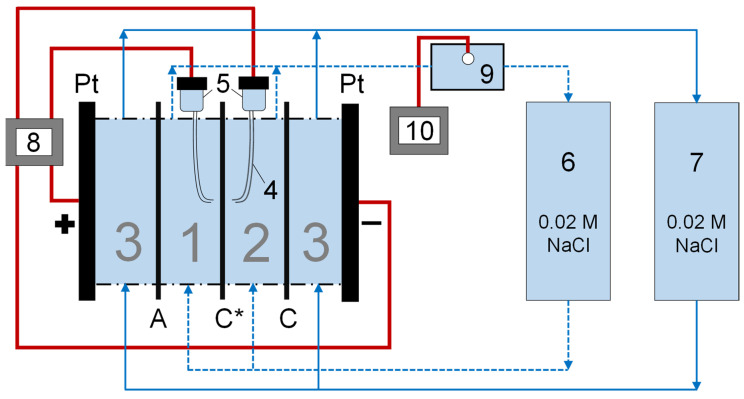
Cell and circuitry for registration of current–voltage curves. Blue lines denote the hydraulic connections and red lines denote electric circuits. Note: 1 is the desalination chamber; 2 is the auxiliary chamber; 3 is the electrode chambers; 4 is the capillaries, connected to 5, which represents Luggin electrodes; 6 is the tank gathering solution from desalination and auxiliary chambers; 7 is the tank gathering solution from electrode chambers; 8 is voltmeter and power supply; 9 is the interstitial tank in the desalination track holding the glass electrode and conductivity cell; 10 is the pH meter and conductometer. Note: C* is a studied membrane; C and A are auxiliary cation exchange and anion exchange membranes, respectively.

Current–voltage curves were recorded using a laboratory flow-through cell (Figure 2) using a four-electrode scheme. Two polarizing platinum electrodes bound the cell from the sides and two Ag/AgCl electrodes were connected to Luggin capillaries located on both sides of the studied sample at a distance of about 1 mm from its geometric center; all electrodes were connected to a current source, an Autolab PGSTAT 100N (Metrohm, Utrecht, the Netherlands) power supply and voltmeter. The measurements were carried out in the galvanodynamic mode with a current density sweeping from 0 to 5 mA/cm^2^ at a sweep rate of 2.5 μA/(cm^2^ s). A 0.02 M NaCl solution circulated through the entire cell, the products of the cathodic reaction were separated from the studied membrane by an auxiliary MK-40 cation exchange membrane, and the products of the anodic reaction were separated by an auxiliary MA-41 anion exchange membrane. The investigated multilayer membranes were arranged in the installation in such a way that the cation exchange substrate was facing the auxiliary chamber; that is, it was oriented toward the cathode and the modifying layers faced the desalination chamber, i.e., were oriented toward the anode. To determine membrane resistance, we also recorded the current–voltage curve without the studied membrane installed, from which we calculated the resistance of the layers of solution between the Luggin capillaries and the membrane surface. The registration of curves was repeated three times for each studied sample.

Current–voltage curves of the MK-40+1 membrane were recorded at a previous stage of the experiment using a slightly different setup than the one used at this stage of the experiment. The difference is that it is easier to account for the slightly longer desalination path of 2.15 cm for MK-40+1 vs. 2.00 cm for all other membranes. There also could be different distances between the tips of Luggin capillaries, so to compare the current–voltage curves of these membranes, we used coordinates that took into account the differing limiting current density and Ohmic resistance values of the solution (namely a dimensionless current density vs. reduced potential drop).

To compare the values of the limiting currents, we determined the experimental limiting current densities of the membranes graphically by finding the points of intersection of the tangents to the initial section and to the plateau section of the current–voltage curve and compared them with each other and with the theoretical limiting current density (theoretical LCD) calculated using the Lévêque Equation [35]:

(1) ilimtheor=z1C1FDh(T1−t1)[1.47(h2V0LD)13−0.2]

where z_1_ and C_1_ are the charge and molar concentration of the counterion in the solution core, respectively; D is the salt diffusion coefficient in the solution (equal to that at infinite dilution and 25 °C, which is 1.61 × 10^−5^ cm^2^/s); h is the intermembrane distance (0.66 cm); T_1_ and t_1_ are the counterion transport numbers in the membrane and in solution, respectively (1, assuming the membrane to be absolutely selective, and 0.603, respectively); V_0_ is the linear solution pumping rate (0.36 cm/s); L is the length of the desalination path (2.15 cm for MK-40+1, 2.00 cm for all other membranes).

The limiting current of Na^+^ through the cation exchange membrane, calculated according to Equation (1) for desalination of 0.02 M NaCl, is 1.96 mA/cm^2^ for MK-40+1 and 1.93 mA/cm^2^ for all other membranes.

The Lévêque equation was derived to describe the one-dimensional case of salt electrodiffusion through a membrane. Since it contains initially known values (except the transport number of the counterion through the membrane, which in the case of dilute solutions and sufficiently selective membranes, is assumed to be equal to unity), it can be used for easy estimation of the limiting current density for a homogeneous membrane. However, it does not consider the generation of H^+^ and OH^−^ ions, additional ion transport mechanisms such as exaltation effect- [36] and current-induced convection [37], or the presence of multilayers. The mathematical description of the current–voltage curve of the multilayer membrane was given by Filippov [38].

The intensity of the generation of H^+^ and OH^−^ ions was estimated from the pH difference between the outlet and inlet to the desalination chamber. For this purpose, additional containers into which glass electrodes were placed connected to Expert 001 pH meters (Econics-Expert, Moscow, Russia) were introduced into the desalination path upstream and downstream of the desalination chamber.

### 2.4. Electrodialysis of Mixed Solution

To study the monovalent selectivity of membranes that can be achieved using the suggested technique, the MK-40+5 membrane performed electrodialysis desalination of a mixed 0.015 M NaCl + 0.0075 M CaCl_2_ solution. The electrodialysis was conducted in a galvanostatic mode using the same cell that was used to register current–voltage curves of MK-40 and MK-40+2 − MK-40+5. The current density was 1.5 mA/cm^2^ (which corresponds to an underlimiting current mode, since the limiting current density of such solution calculated by the Lévêque equation is 2.7 mA/cm^2^ and the registered current–voltage curve given below shows that the experimental limiting current densities are comparable to the theoretical ones or higher than them) and 100 mL of solution initially filled the desalination tract. Desalination was carried out for 5 h (18,000 s); each hour, the pH value of the sampled solution was registered and an aliquot 1 mL in volume was taken to determine the contents of Na^+^ and Ca^2+^ using an Akvilon A-2 (Akvilon, Moscow, Russia) atomic absorption spectrometer.

## 3. Results and Discussion

Let us state right away that we can speak about the stability of the resulting coating only within the framework of the current–voltage curves recorded before and after an hour of working under current densities up to 5 mA/cm^2^ and with comparison to works that studied the stability of such adsorbed polyelectrolyte layers. More thorough studies of stability would make a very interesting topic for future investigations.

### 3.1. Thickness Measurement

**Figure 3:**
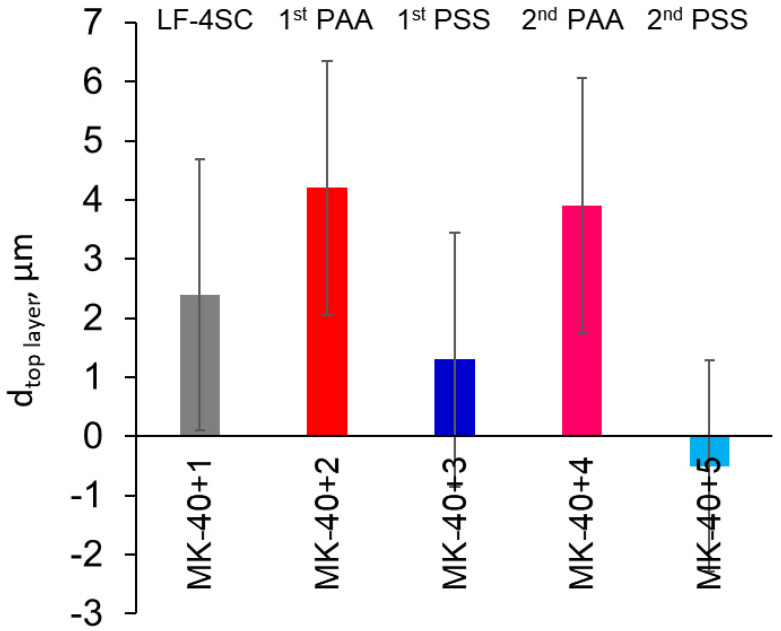
Increments of thickness of modified membranes in comparison with membranes with one less adsorbed layer. Error margins are sums of confidence intervals calculated for ten repeated measurements for these two membranes at α = 0.05. MK-40 is a heterogeneous cation exchange membrane, MK-40+1 is a MK-40 coated with homogenizing layer of perfluorsulfonic acid, MK-40+2 is a MK-40+1 with an additional adsorbed layer of polyallylamine, MK-40+3 is a MK-40+2 with an additional adsorbed layer of sodium polystyrene sulfonate, MK-40+4 is a MK-40+3 with an additional adsorbed layer of polyallylamine and MK-40+5 is a MK-40+4 with an additional adsorbed layer of sodium polystyrene sulfonate. Text denotes the composition of the top layer (LF-4SC for perfluorosulfonic acid, PAA for polyallylamine and PSS for sodium polystyrene sulfonate) and its position in a row of layers of the same nature, numbered from the first one applied to the last.

To determine the thickness of polymeric layers, we deduced the thickness of the membrane with one less absorbed layer from the membrane with one more absorbed layer. The results of the calculations are given in Figure 3. It can be seen that not taking into account the densification of previous layers, the PAA layers are 4.2 ± 2.2 µm and 3.9 ± 2.2 µm thick, while regarding the PSS layers, it can only be claimed that they are below 3.5 µm in thickness. Let us also note here that the substrate membrane used in these experimental runs was 555 µm thick, while the PEI layer of samples created at previous stages of investigation using the same technique was 12 ± 4 µm thick.

Layer-by-layer polyelectrolyte assemblies are often cited as being very thin [39], and there are works that report that polyallylamine–polystyrene sulfonate coatings have nanometer-scale thickness [40]. Our assemblies, both the polyethyleneimine–polystyrene sulfonate tested in the previous article and polyallylamine–polystyrene sulfonate tested in the present work, were several orders of magnitude thicker than this. It can surely be concluded that more than one monolayer of polyelectrolyte was adsorbed at each stage. In our technique, the adsorption time was higher than normal for the creation of PAA–PSS multilayers (we dipped the membranes for 1 h, while other studies dipped them for 5 min [41], 20 min [42], and 30 min [21,43]). Higher thickness cannot, however, be discounted for greater adsorption, since it was shown that adsorption of these polyelectrolytes asymptotically approaches the constant value with time and for PAA–PSS systems the increment in adsorption past those 15–20 min is not that big [40]. Another study showed that the thickness of layer-by-layer polyelectrolyte assemblies may be increased due to the presence of defects, such as pockets of electroneutral solution or coacervates [44]. We can hypothesize that the increase in thickness might be caused by the formation of defects such as these. 

### 3.2. Current–Voltage Curves

**Figure 4:**
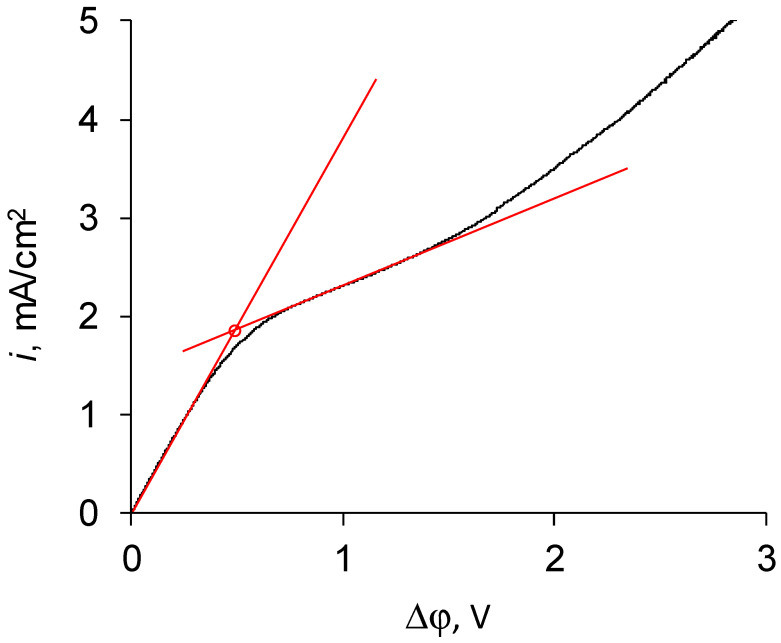
Determination of the experimental limiting current of the MK-40 membrane by the graphical method.

Figure 4 shows the current–voltage curve of a commercial MK-40 membrane and shows a graphical method for determining the experimental limiting current density by drawing tangents to the so-called Ohmic section and to the plateau section.

**Figure 5:**
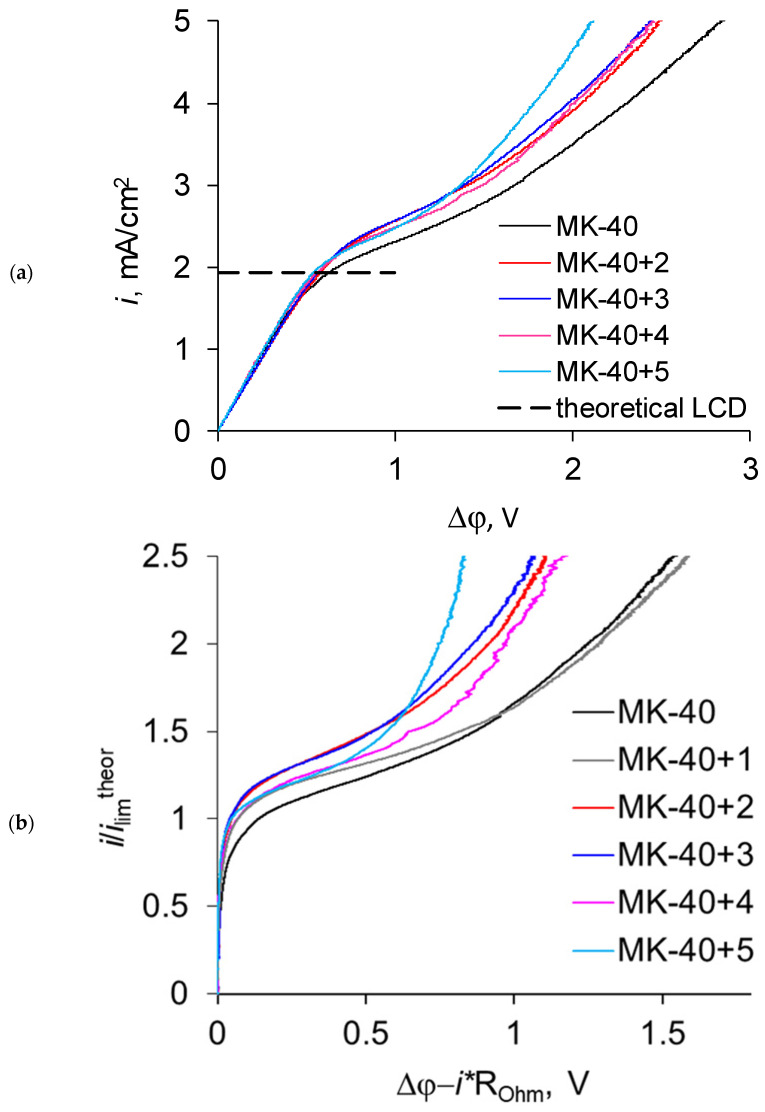

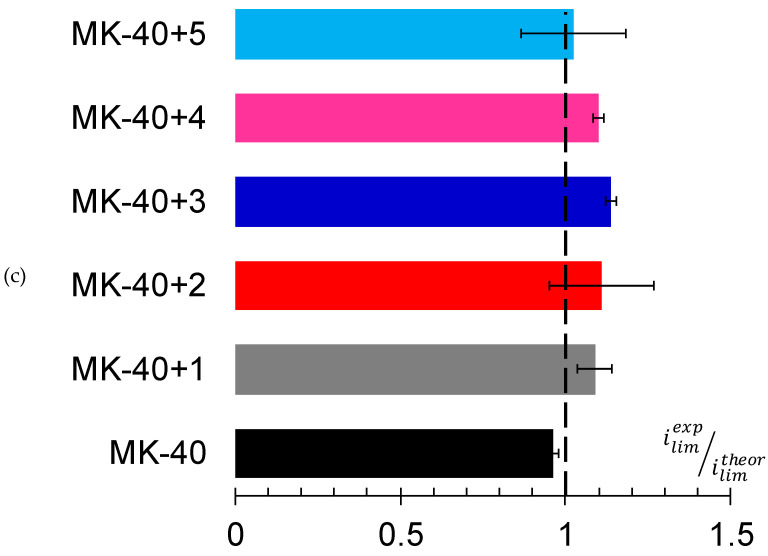
(**a**) The current–voltage curves of the MK-40 membrane and layer-by-layer-modified samples based on the membrane (sans MK-40+1), created using PAA. (**b**) The current–voltage curves of all studied membranes in “dimensionless current density vs. potential drop reduced by Ohmic component” coordinates, allowing comparison between different cell geometries. (**c**) The experimental limiting currents of these membranes related to the theoretical limiting current density calculated by the Lévêque equation. The dashed line shows the theoretical limiting current density calculated by the Lévêque equation.

Figure 5 shows a comparison of current–voltage curves of the original MK-40 membrane and modified membranes based on it. Figure 5a gives the curves of MK-40 and MK-40+2–MK-40+5 membranes with the following coordinates: current divided by polarized area vs. potential difference between Luggin capillaries, which provide more information about the system but are harder to compare between different cell geometries, since the slope of the curve depends on the Ohmic resistances of the membrane and the solution between the capillaries, and hence on the distance between capillaries. Therefore, to see the curve for MK-40+1 (which was recorded using different cells) and the curves for the other membranes in one figure and for easier comparison between different cell geometries, we present Figure 5b, which is built with different coordinates. The abscissa is the reduced potential drop, where we subtract the current density *i* multiplied by the Ohmic areal resistance of the membrane and the solution between the capillaries *R*_Ohm_ (determined as the ratio of the potential drop at the so-called Ohmic region of the current–voltage curve to the current density) from the total potential drop between the Luggin capillaries Δφ. The ordinate is the dimensionless current density, where we divide the experimental current densities by the theoretical limiting current density calculated using the Lévêque equation.

It can be seen that the curves are similar and the graphically determined experimental limiting current densities are close to each other and to the value of the theoretical limiting current density calculated using the Lévêque equation for a single-layer membrane. This means that application of layers of polyelectrolyte does not significantly reduce the limiting current density, as occurs with asymmetric bipolar membranes [24]. The absence of a difference after application of the first such layer might be explained by its thickness being lower than necessary to block the transport of counterions. However, multiple layers become comparable in thickness with such layers in the asymmetric bipolar membrane. It might be suggested that some of the formed bipolar boundaries did not fully block or had defects that allowed Na^+^ ions to pass.

There are several things that could appear on the current–voltage curves of membranes modified with polyelectrolytes with alternating charges of fixed groups, as outlined below, however they are absent in the recorded curves.

**Figure 6:**
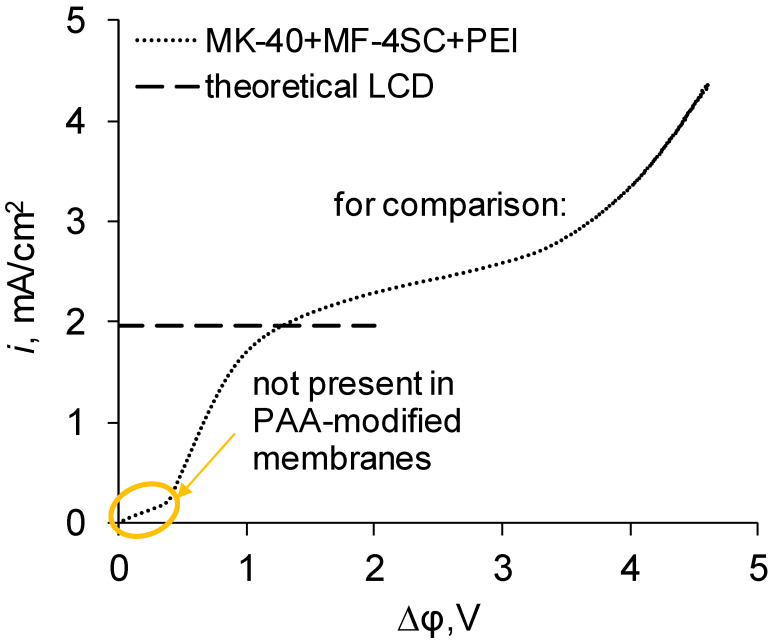
The current–voltage curve of the MK-40 membrane modified with MF-4SC and PEI, given for comparison. The dashed line shows the theoretical limiting current density calculated by the Lévêque equation. LCD denotes the limiting current density, PEI denotes polyethyleneimine and PAA denotes polyallylamine.

First, there could be a pattern typical of a bipolar membrane and the curve could have a sharp increase in the potential drop in the initial section due to desalination of membrane layers followed by a sharp increase in current density at almost constant potential drop later due to the onset of intensive generation of H^+^ and OH^−^ ions [24]. We observed similar pattern in our previous work, in which PEI was chosen as a modifier (see Figure 6); the initial section was present but it was short in comparison with values typical for a bipolar membrane, and further growth of the current density was achieved not through appearance of new charge carriers, but through the counterions moving through a layer with the same charged fixed groups. The existence of this section would be a problem when applying the modified membranes, since it moves the entire curve to higher potentials, meaning higher energy demands for apparatuses operating in galvanostatic mode or lower performance for apparatuses operating in potentiostatic mode, and for electrodialysis fractionation or purification it would be desirable to eliminate this section. We expected that this section would be present in the curves of new samples, since we considered PAA to be similar to PEI in terms of formation of the layer carrying positively charged fixed groups, and we also expected that its length would be somewhat changed as a result of two factors—either it would be lengthened due to the appearance of new bipolar boundaries or it would be shortened due to the lesser basicity of PAA in comparison with PEI and the subsequent weaker electrostatic repulsion of counterions. In reality, the current–voltage curves of the new samples do not have the initial section containing the rapid increase of electric potential. While this information makes new membranes more suitable for future applications in electrodialysis purification, it raises the question of the origin of this difference. For now, we can suggest a hypothesis that the existence of this zone strongly depends on the thickness of the blocking layer, whereby a PAA layer of about 4 µm in thickness blocks the transport of cations much more than a PEI layer of about 12 µm in thickness.

**Figure 7:**
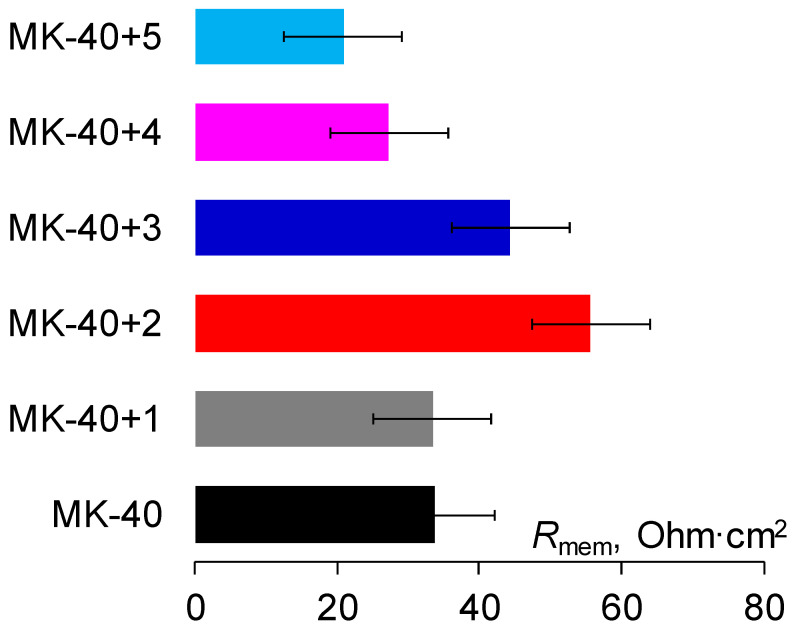
The areal resistance values of the membranes in 0.02 M NaCl solution, calculated from initial sections of the current–voltage curves using the difference method.

Second, the application of layers could significantly increase the electrical resistance of the system. We expected that the addition of each layer would increment the resistance due to the formation of additional bipolar boundaries, and that these resistance increases would be comparable to the very first increase of resistance due to application of the PEI layer during the formation of MK-40+2. To evaluate the membrane resistance at operating concentrations: (1) we referred to readings of potential drops between Luggin capillaries and of current densities recorded when registering the current–voltage curves of the fully assembled cells and the cells without the studied membranes installed; (2) we calculated the Δ(Δφ)/Δ*i* for initial sections of curves to obtain the areal resistance *R* values of two solution zones with a membrane between them and a solution without the membrane installed *R*_sol_; (3) we subtracted *R*_sol_ from *R* to obtain the areal resistance of the membrane *R*_mem_. The calculated areal resistance values of the membranes are given in Figure 7.

It can be seen that the differential areal resistance of the membranes increased after application of the first PAA layer, however with the application of the subsequent layers it started to decline. The negative differential resistance of the polyelectrolyte layers means that new applied layers reduce the resistance of other parts of the system. This might occur due to two processes. To explain the first one, let us refer to the construction of the cell and the membrane modification procedure. During the modification, the polyelectrolyte is adsorbed only in the central zone of the membrane, which later becomes polarized. This means that the polyelectrolyte is not present at the edges of the membrane that contact the frames that hold the Luggin capillaries, and hence the distance between the capillaries does not grow due to adsorption of layers. Hence, the polyelectrolytes partially replace the solution between the Luggin capillaries and the observed decrease is at least partly attributed to artifacts of the difference method used for determination of resistance. It can be seen from the calculated resistance values that the resistance of 0.02 M NaCl is much higher than the resistance of the membrane and layers (the areal resistance of the solution is about 222 Ohm cm^2^ vs. 15–60 Ohm cm^2^ for membranes), so this replacement would decrease the resistance. The effect of the higher conductivity of polyelectrolytes is illustrated by membranes MK-40+4 and MK-40+5, which have the same (within the confidence interval) thickness and almost the same resistance.

It does not, however, explain the absence of resistance growth with the creation of additional bipolar boundaries. When the PAA layer was applied to the MK-40+1 membrane to create MK-40+2 and to form the very first bipolar boundary, it increased both the membrane thickness (hence displacing part of the solution between the capillaries) and the resistance. It could be concluded that in the balance of effects that determine the resistance, the effect of the creation of the bipolar boundary outweighs the effect of the displacement of solution. However, for all consequent applied membranes, the application of a new layer leads to a net loss of resistance, suggesting that the effect of the displacement of solution is greater than the effect of the creation of new bipolar boundaries.

It seems to us that the lack of resistance growth is related to an almost constant limiting current density, and the common origin is in the created bipolar boundaries not being equal. It might be hypothesized that the additional bipolar boundaries and layers charged oppositely to transported counterions do not block the first bipolar boundary or the first oppositely charged layer. 

The third membrane property that could be expected is the early onset of the generation of H^+^ and OH^−^ ions due to the appearance of bipolar boundaries. Let us consider the processes affecting the pH of the treated solution in more detail.

The balance between the generation of these ions in cation exchange and anion exchange membrane determines the shift of pH for the treated solution—protons produced in cation exchange and hydroxyls produced in anion exchange membranes leave the desalination chamber, while hydroxyls produced in cation exchange membranes and protons produced in anion exchange membranes are (mostly) retained, so if generation of H^+^ and OH^−^ ions is more intensive in cation exchange membranes, the solution becomes alkalified and the pH increases, while if this generation is more intensive in anion exchange membranes, the solution becomes acidified and the pH decreases. In systems with monopolar membranes, the onset of the noticeable generation of H^+^ and OH^−^ ions normally occurs when the concentration of salt counterions decreases during desalination and reaches a critically small value. If the mobility of the cations and anions in the solution is not equal, then the generation initially starts in the membrane counterions, which have lower mobility in solution. When the limiting state is reached in the solution near both membranes, then the shift of pH is determined by the rates of these reactions, which in turn depend on the catalytic activities of fixed groups, which are known beforehand [25]. The situation becomes more complicated in solutions of salts that can dissociate.

We used a NaCl solution, as the mobility of Na^+^ is lower than the mobility of Cl^−^, hence we expected that the limiting state would first be reached near cation exchange membrane and initially the solution would be alkalified. Since the bipolar boundary tends to boost the generation of H^+^ and OH^−^ ions and the membrane substrate does not contain bipolar boundaries while the modified membranes do, we expected that at low currents MK-40 and MK-40+1 would demonstrate weak alkalinization (about 0.1 units of pH) and the modified membranes would demonstrate slightly stronger alkalinization (based on previous experience, about 0.2 units of pH). The cation exchange membrane contains −SO_3_^−^ groups that have low catalytic activity in terms of generating reactions of H^+^ and OH^−^ ions and the anion exchange membrane has −N^+^R_3_ groups, which are created during its synthesis, as well as −N^+^R_2_H and −N^+^RH_2_ groups as a result of degradation of initial quaternary ammonium groups; the quaternary ammonium group has low catalytic activity in the generation of H^+^ and OH^−^ ions and the amino groups have high catalytic activity in this reaction, so we expected that after the limiting current is reached, the alkalinization would be changed to acidification.

**Figure 8:**
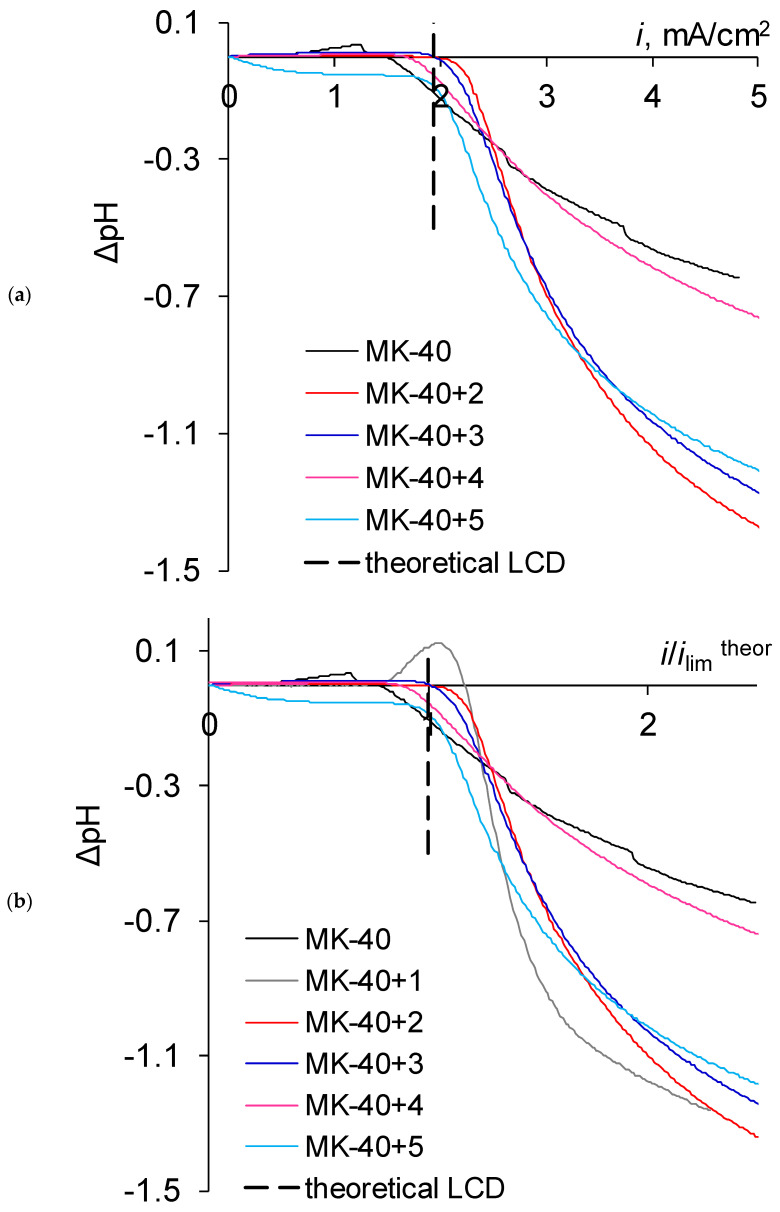
Change in pH of the treated NaCl solution after passing through the desalination chamber: (**a**) dependence of pH on current density for all membranes except MK-40+1; (**b**) dependence of pH on dimensionless current density *i*/*i*_lim_^theor^, whereby i_lim_^theor^ was calculated using the Lévêque equation. LCD denotes the limiting current density.

The results for the pH differences are given in Figure 8.

As can be seen, in underlimiting current modes the pH shift is quite weak, even for the substrate membrane, for which the highest registered pH shift was 0.015. The shifts were even lower for MK-40+1, MK-40+2, MK-40+3, and MK-40+4. For MK-40+5, the shift of pH was greater, e.g., at 0.8 *i*_lim_^theor^ the pH shift was –0.054, however for the studied orientation of the membrane and the modifying layers in the cell, the increased generation of H^+^ and OH^−^ ions would lead to alkalinization instead of acidification.

The magnitude of the registered pH shifts lets us claim one thing for certain—the proposed technique does not cause intensive generation of H^+^ and OH^−^ in the studied systems in underlimiting current modes. Due to the same low magnitude of the observed shifts, the below conclusions are more speculative.

This small pH shift is not the most typical observation for commercial MK-40, however we have registered similar values before, which can be explained by the properties of the batch. We can assume that the batch from which these samples were obtained had a higher surface fraction of ion exchange resin, hence having a lower local current density and a lower local concentration polarization.

Since in all experiments we used the same paired anion exchange membrane, the decrease of pH in the treated solution with the growing number of applied layers at the same current densities was caused by the lower intensity of generation of H^+^ and OH^−^ ions in the cation exchange membrane. We can speculate on two possible mechanisms for this change. The first one is homogenization of the surface; while only about 20–30% of the surface of the substrate membrane is conductive, the application of polyelectrolytes makes the entire surface conductive, decreasing the local current density and reducing the concentration polarization. The second is the barrier effect of polyelectrolytes for transport of the generated protons through positively charged PAA and, which then would not leave the desalination chamber but would neutralize the generated hydroxyls, suppressing the alkalinization. The observation of stronger acidification for the MK-40+5 membrane in comparison with the other samples might be explained by the combination of weak catalytic activity of sulfonic groups on its surface in the generation reaction of H^+^ and OH^−^ ions and strong blocking of proton transport by inner layers of PAA. It would be interesting to see if MK-40+6 demonstrates acidification and MK-40+7 demonstrates even stronger alkalinization.

From the perspective of changes occurring in membrane properties, let us consider an earlier work in which the MK-40 membrane was modified to decrease its hydrophilicity and boost its limiting current and overlimiting mass transfer due to electroconvection [45]. That study used the same solution and a very similar experimental setup, while the ratios of the experimental limiting current density to theoretical limiting current density calculated by the Lévêque equation were in the range 0.9–1.2. The same ratios for modified membranes reported in the present work are in the range 1.0–1.15, so we can consider the modification to be successful, even in terms of improving the limiting current density. Since almost no shift of pH was observed in underlimiting current modes at which electrodialyzers usually operate, we also consider the modification to be successful in terms of undesirable generation of H^+^ and OH^−^ ions.

### 3.3. Electrodialysis of Mixed Solution: Evaluation of Monovalent Selectivity

**Figure 9:**
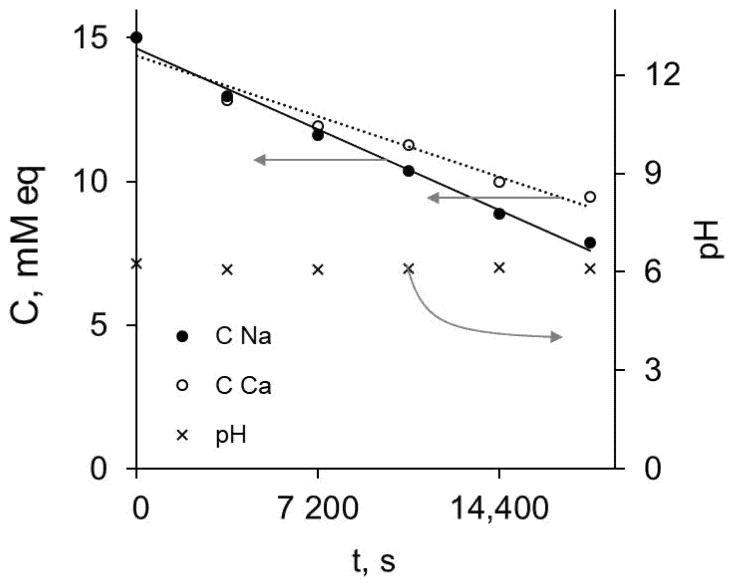
Concentration changes for cations during electrodialysis desalination of a 0.015 M NaCl + 0.0075 M CaCl_2_ solution in a system with a MK-40+5 modified membrane at constant *i* = 1.5 mA/cm^2^. The theoretical limiting current density is estimated as 2.7 mA/cm^2^.

Figure 9 shows the determination results for concentrations of sodium and calcium in samples taken during electrodialysis desalination of the mixed solution (0.015 M NaCl and 0.075 M CaCl_2_) and the pH values registered at the outlet of the desalination chamber. The first point of concentration for the salt ions is 15 mM, corresponding to the concentrations of introduced salts in the solution that initially filled the desalination tract.

The commercial MK-40 membrane more readily transports polyvalent ions (as shown by Mishchuk et al. [46]). It can be seen that for the modified membranes, the concentration of sodium decreases faster than the concentration of calcium, so the modified membrane is monovalent-selective. The achieved selectivity can be calculated by the equation adapted from [15]:

(2) $$P_{Ca^{2+}}^{Na^{+}}=\frac{J_{Na^{+}}C_{Ca^{2+}}}{J_{Ca^{2+}}C_{Na^{+}}}$$

where P_Ca^2+^_^Na^+^^ is the monovalent selectivity of the membrane; J_Na^+^_, J_Ca^2+^_ and C_Na^+^_, C_Ca^2+^_ are fluxes of ions through the modified membrane and concentrations of ions in the desalination chamber, respectively.

The fluxes can be easily calculated from the linear approximation of the dependence of the concentration on time and the known volume of the solution in the desalination tract.

The resulting monovalent selectivity values are in the range of 1.33–1.56, which is rather modest in comparison with the best findings reported for the layer-by-layer approach, which exceed 1000. This might be in part due to the lower number of applied layers (5.5 bilayers in [12] vs. 2.5 bilayers in our work) and the other mechanisms, such as the presence of defects within the layers or the lower exchange capacity due to swelling. It should be noted that the selectivity obtained in our work is comparable to the values provided in [15] for special commercial-grade membranes, where P_Ca^2+^_^Na^+^^ values were found to be 1.23 (Neosepta CMS) and 1.72 (Selemion CSO).

## 4. Conclusions

Layer-by-layer-modified membranes based on the heterogeneous MK-40 membrane, which use polyallylamine and polystyrene sulfonate as modifiers with alternating fixed group charges, demonstrate that the electrical resistance grows after application of the first polyallylamine layer, but with increasing numbers of layers becomes comparable to that of the substrate. The experimental limiting current density of the modified membranes is higher and the pH shift of the treated solution is low in magnitude and comparable with that of the substrate membrane. The achieved values for the limiting current density are comparable with the results obtained earlier for an MK-40 sample specially modified to improve the mass transport of salt ions through this membrane.

The electrodialysis of the mixed NaCl + CaCl_2_ solution showed that the used approach creates monovalent selectivity values in the range of 1.33–1.56, which is comparable to commercial membranes but far from the highest values reported for the layer-by-layer approach.
